# Design of efficient binary multiplier architecture using hybrid compressor with FPGA implementation

**DOI:** 10.1038/s41598-024-58482-0

**Published:** 2024-04-11

**Authors:** V. Thamizharasan, V. Parthipan

**Affiliations:** 1 Department of ECE, Erode Sengunthar Engineering College, Erode, India; 2 Department of ECE, Sri Eshwar College of Engineering, Coimbatore, India

**Keywords:** Hybrid, Multiplier, Compressor, Xilinx, Field programmable gate array (FPGA), Spartan6, Engineering, Mathematics and computing

## Abstract

In signal processing applications, the multipliers are essential component of arithmetic functional units in many applications, like digital signal processors, image/video processing, Machine Learning, Cryptography and Arithmetic & Logical units (ALU). In recent years, Profuse multipliers are there. In that, Vedic multiplier is one of the high-performance multiplications and it is used to signal/image processing applications. In order to ameliorate the performance of this multiplier further, by proposed a novel multiplier using hybrid compressor. The proposed hybrid compressor-based multiplier is designed and implemented in Field programmable Gate Array (FPGA—spartan 6). The synthesis result shows that the speed of proposed hybrid compressor-based multiplier gets improved as compared to Array multiplier (35.83%), Wallace tree multiplier (34.58%), Vedic Multiplier based on Carry look ahead adder (CLA) (28.49%), Vedic Multiplier based on Ripple carry adder (RCA) (20.65%), Booth Multiplication (21.65%) and Vedic Multiplication based on Han-Carlson Adder (HCA) (20.10%) and Hybrid multiplier using Carry Select Adder (CSELA) (17.81%) and Hybrid Vedic Multiplier (7.15%).

## Introduction

In the smart life Digital era, Multimedia, Cryptography, Artificial Intelligence, Machine Learning, Deep Learning, Internet of Things (IoT) and 4G/5G Technology are going to play a crucial role^1^. In these technologies, they involve huge basic arithmetic calculations, such as multiplication and addition with a huge volume of data^2^. In that, the power utilization, area utilization and delay are the very important parameters to decide the entire system performance. The optimization of power consumption and area is great demand and very challenging assignment in High performance multipliers and adders^3^.

There are various multiplier architectures available based on the major classification of serial multiplier, parallel multiplier and serial/parallel multiplier. Also, various algorithms are available to implement the high-performance multiplication operation in the literature. Similarly various adders are available in the literature. They are classified based on the rippling of intermediate carry. Consequently, it is essential to scrutinize the utilization of area, power and speed of the multiplication and addition functional unit in the signal processing applications.

The pivot of the presented work is designing a high-speed multiplier using hybrid compressor. The showcased model modifies the multiplier architecture using 12:6 and 5:3 hybrid Compressor and 3:2 compressor and it implemented in FPGAs. The results claimed that optimal Speed, retaining architecture of parallelism as compared to its high-performance variant.

The paper is systematized as follows. The existing adders is presented “Review of adders” section. In “Proposed multiplier” section, the existing multipliers are described. In “Performance analysis” section, the proposed hybrid compressor-based multiplier is projected. In “Conclusion” section, the experimental results are compared and analyzed with existing technique. Finally, “Conclusion” section provides the conclusion of this article.

## Review of adders

In digital Integrated Circuits (IC) design, addition operation is the brain of arithmetic module. It has special significance in portable devices, compactible devices, cryptography, Artificial Intelligence, Machine Learning, signal, audio, image and video processing^4^. The above applications require multi-bit adders and produce high delay due to rippling of long carry propagations are the great issue in adders. Hence, there is a need in designing of high speed and low complexity architectures of adder^5^.

The ripple carry addition has less power utilization and occupies minimum area as compared to most of the adder architectures. But it will linearly increase the delay in bit size of the adder makes and it will be unsuitable for a high-speed application^6^.

In carry save adder, the 3-bits are added simultaneously and carry are stored in present stage and not propagated through the subsequent stages. The speed of this adder is improved due to carry generation. The pros of this adder are adding three input values at a time. However, cons of this adder are occupied large area due to more number of transistor and consume large power.

There are few high-speed adders available in literature such as carry skip adder, the carry look ahead adder (CLA), conditional adder, carry select adder (CSELA) and their combinations.

Carry Look Ahead Adder (CLA) is one of the fast adders. Because, based on generation and propagation principle the sum and carry generation can be done at same time. Here the output carry depends on only input carry irrespective of the bit size. The pros of this adder are lees delay as compared to Ripple Carry adder (RCA). However, cons of this adder are occupied large area due to separate circuit for sum and carry generation. Also, the complexity of the circuit increases with increase bit size^7^.

Another one of the fastest adder is Carry select adder. This adder can perform the addition operation based on the pre assumption of input carry (Assume input carry Cin = 1 or 0). This adder can be improved the speed as compared to Ripple Carry adder (RCA) and Carry Look Ahead Adder (CLA), but it is consumed more area due to dual Ripple Carry adders (RCAs).

To reduce the size of Carry Select adder (CSELA) the binary to excess one conversion (BEC) is introduced. The Modified Carry Select adder (CSELA) designed by the second Ripple Carry adder (RCA) (for Cin = 1) is replaced by Binary to Excess one Converter (BEC). This Binary to Excess one Converter (BEC) occupied the less numbers of Gate (transistor) as compared to the Ripple Carry adder (RCA). Hence the area is reduced^7^.

It is further improved the delay of the Carry Select adder (CSELA) by introducing the concept of parallel prefix adder. It is named as Brunt Kung Adder (BK adder). The delay of circuit is improved by modifying the first stage by BK adder (Replacing RCA for Cin = 0 with BK adder) and second stage by Binary to Excess one Converter (BEC) (Replace RCA for Cin = 1 with BEC)^8^.

Furthermore, the utilization of area by Common Boolean logic (CBL) was developed. In this logic, the existing resource will be utilized to minimize the number of gates. In Carry Select adder (CSELA), the RCA for Cin = 1 is replaced by CBL^8^.

For optimizing the speed and area of CSELA addition Han-Carlson adder is further introduced. This adder is designed on the features of Kogge stone and Brent Kung adder (BK). Since Koggestone have a less delay and Brent Kung has a less area utilization. The combination of BK adder (For Cin = 0) and Koggestone adder (for Cin = 1) is named as Han Carlson adder^9^.

To ameliorate the speed of operation by introducing the parallel carry computation in addition is called as Weinberger recurrence algorithm. In Han-Carlson adder (HCA), the BK adder (for cin = 0) is changed by the modified BK adder and Koggestone adder (for cin = 1) is changed by the 5 bit Binary to Excess one Converter (BEC) module^9^.

### Review of multipliers

In numerous applications, multiplication is one of the prominent components which conquest the overall performance of the Signal processing system. Multiplication is a key arithmetic operation, that has a most important primitive component in the performance of various digital processors, machine learning, internet of things, deep learning, Finite impulse response (FIR) filters, convolution, Fast Fourier Transform (FFT), distributed computing, Arithmetic and Logic Unit (ALU), signal/Image processing, and multimedia applications. It decides the area, delay, and overall performance of parallel implementations^10^.

In signal processing application devices, such as smart phones, Laptop, tablet, and Personal computers are required high performance multiplier with the importance of minimum area utilization is very important one. There are various methods available to implement the multiplication operation.

Multiplier are mainly categorized by two methods such as serial and parallel multiplication. In that, serial multiplication, every bit of multiplier is used for calculating the partial products. While parallel multiplication, the partial products of every bit of multiplier are calculated in parallel. The performance (speed) of the multiplication mainly depends on the generation of partial products. The speed is optimized in parallel implementation of multiplication with penalty of area utilization^11^.

In multiplication operation, the bit by bit AND operation is followed by addition of partial products with the help of half and full adders. Speed of a multiplication mostly depends on the number of partial products generations and accumulation.

The multipliers are categorized based on reduction of partial products. There are Array multiplication, Wallace multiplication, Bypassing multiplier, Booth multiplication, Vedic multiplication and Booth recorded Wallace tree multiplication, Baugh Wooley multiplication, Braun multiplication and etc.,^12^.

Approximation gives an alternate technique to optimizing the accuracy of multiplication without compensating another circuit.

In addition to the above multiplication, the truncated multiplication is a highly specialized type of multiplication that only determines the part of the product. Because, Approximate calculation is the best technique for an error tolerant and energy efficient applications, that exhibition of essential tolerate the erroneousness, such as signal processing and multimedia applications. Approximate computing was reduced the accuracy of multiplication, nevertheless it still provides a faster result with less power consumption, this method was used in some part of arithmetic circuits in signal processing applications.

A various arithmetic architecture was designed using exact and deterministic principles. But, many applications, namely multimedia, signal/image processing can allow/permit the errors and generate results which are better for human perceptions. Since exact solutions are enough in these error-tolerant application to allow the computing systems to maintain quality and accuracy of the design. Hence it is necessary to concentrate, analyze and investigate the approximate additions and multiplications.

### Review of compressor

Multipliers are omnipresent and essential arithmetic module in Very Large-Scale Integration VLSI architectures, particularly in signal processing applications and general-purpose processors. The speed and power utilization of multipliers are the main parameter to determine the performance of system. The optimum performance of multiplication will be achieved on the minimization of partial products generation during the multiplication process. The efficiency of multiplication can be optimized by minimizing the number of partial products within limit of power consumption. Hence, the compressors are specifically designed as an arithmetic module for optimizing the speed and area of an arithmetic module^13^.

There are various methods extensively applied to minimize the partial products such as recording technique, Booth Recorder, Wallace tree multiplication through carry save adder, Dadda multiplication using modified Wallace tree design and Carry Save Adder.

#### Compressor

The compressor is an important module and it is mostly used in Very Large-Scale Integration (VLSI) circuits and systems and their applications. It is commonly used as a processing element.

A (m, n) compressor consists of m-bit input with carry inputs ‘Cin’ and produces n-bit output with carry output Cout. The main advantages of compressors are producing the output without rippling of carry. Because of Cout is not depended on the input carry Cin. Also, horizontal and vertical signal paths of compressors are simple and regular structure than other existing technique^14^.

#### 3:2 compressor

The most common and simple compressor is 3:2 compressor and it is called as full adder. It is defined as single bit adder and it consists of three inputs and two outputs. Also, it is designed with many Very Large-Scale Integration (VLSI) logic circuit design technique. It mainly consists of the three modules. The 1st module is to compute XOR / XNOR operation. The 2nd module is to determine the output ‘Sum’. The 3rd module is a to evaluate the carry output signal. Full adder can be constructed with the help of these three modules.

The general equation for 3:2 Compressor is

1 $${\text{A }} + {\text{ B}} + {\text{ C}} = {\text{ Sum}} + {\text{ Carry}}\left( {{2} - {\text{bit}}} \right)$$

where

A, B and C are inputs.

Sum and Carry are outputs.

**Figure 1:**
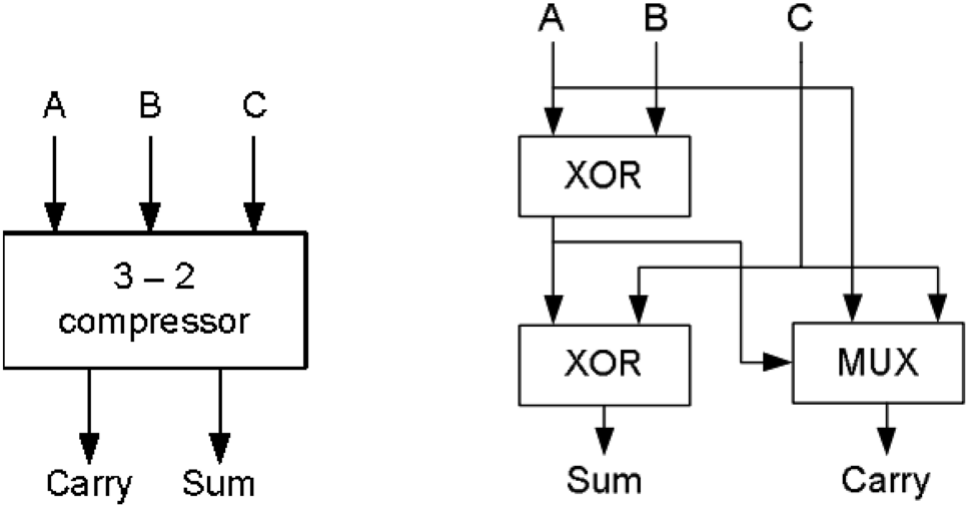
Architecture of 3:2 compressor^14^.

The block diagram and structure of 3:2 compressor is displayed in Fig. 1^14^. This structure consists of 2-Exclusive OR gates in critical path. The output ‘sum’ is computed from 2nd XOR gate and carry is produced by the multiplexer (MUX)^13^. The output equations for 3:2 compressor is

2 $${\text{Sum }} = {\text{ A XOR B XOR C}}$$

3 $${\text{Carry}} = \, \left( {\text{A XOR B}} \right) \cdot {\text{C}} + \left( {\text{A XOR B}} \right)^{\prime } \cdot {\text{ A}}$$

The 3–2 compressor has minimum delay as compared to conventional full adder. In order to improve the speed of compressor, XOR gate is replaced by Multiplexer. In that selection input of multiplexer (MUX) is available before the input signal arrives. Hence, it minimizes the delay of the circuit due to reduction of switching time in critical path of transistor. This will reduce the significant amount of delay. The modified 3:2 compressor output equation is

4 $${\text{Sum}} = \, \left( {\text{A XOR B}} \right) \cdot {\text{C}}^{\prime } + \, \left( {\text{A XOR B}} \right)^{\prime } \cdot {\text{ C}}$$

5 $${\text{Carry}} = \, \left( {\text{A XOR B}} \right) \cdot {\text{C}} + \, \left( {\text{A XOR B}} \right)^{\prime } \cdot {\text{C}}$$

#### 4:2 compressor

**Figure 2:**
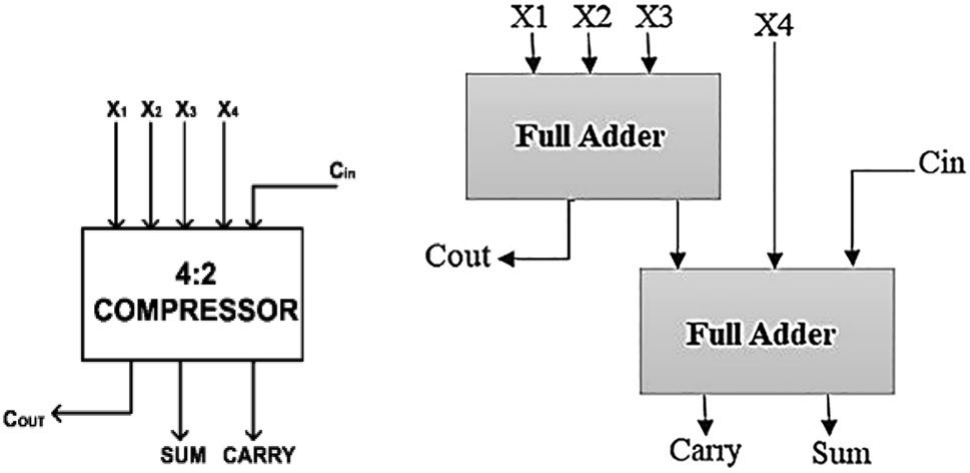
Structure of 4:2 compressor^13^.

Generally, 4:2 compressor is a combination of pair of full adders. The general structure of 4:2 compressor is shown in Fig. 2. It accepts 4 inputs with one carry inputs and compress the two outputs namely ‘sum’ and ‘carry’. It also generates the intermediate carry bit ‘Cout’.

The relation between inputs and outputs of 4:2 compressor is

6 $${\text{X1}} + {\text{X2}} + {\text{X3}} + {\text{X4}} + {\text{Cin}} = {\text{ Sum}} + {\text{ Carry }} + {\text{ Cout}}.$$

where

7 $${\text{Sum }} = {\text{ X1}} \oplus {\text{X2}} \oplus {\text{X3}} \oplus {\text{X4}} \oplus {\text{Cin}}$$

8 $${\text{Cout }} = \, ({\text{X1}} \oplus {\text{X2}}){\text{ X3 }} + \, ({\text{X1}} \oplus {\text{X2}}){\text{X1}}$$

9 $${\text{Carry }} = \, ({\text{X1}} \oplus {\text{X2}} \oplus {\text{X3}} \oplus {\text{X4}}){\text{ Cin }} + \, ({\text{X1}} \oplus {\text{X2}} \oplus {\text{X3}} \oplus {\text{X4}}){\text{ X4}}$$

A 4:2 compressor is generally designed by a combination of multiplexers and XOR gates. The 4:2 compressor was designed by simply cascading of 2 full adders and it is shown in Fig. 2^13^. It achieves the critical path delay of 3 XOR gate delay^14^.

In order to optimize the performance of the 4:2 compressor by design of multiplexer using full swing pass transistor logic to achieve optimized power utilization. The pros of this design are sum generation not dependent on Cout generation, as compared to 4:2 compressor structure shown in Fig. 2. Also, this design was achieved 18% delay improvement as compared to conventional full adder design.

Further it improves the speed of operation, proposed a modification of compression unit by rearranging the Boolean equation to improve the delay of carry computation. The modification of compression unit was designed by combined a NAND and NOR gate into an XOR gate.

For optimizing the performance of the arithmetic circuits by rearranging the Boolean/logic equation or derive the new logic equation from the truth table. The change of Multiplexer against with XOR logic in right places of the existing technique is implemented through Shannon’s expansion technique^15^.

The performances are mainly determined by their speed of arithmetic calculation. Arithmetic computation Adders, Shifters and Multipliers are the crucial module of any Signal Processing applications. In addition, process due to huge carry computation delay and sequential behavior, existing digital system architecture is slow in nature.

Also, the Multiplication operation determines the speed of the most Digital Signal Processing (DSP) applications, hence it required high-speed multiplier for an efficient data path circuit design. In order to improve the speed of a multiplier, it minimizes the number of the partial product, since multiplication leads to series of addition of partial products.

## Proposed multiplier

The major constraint of the multipliers are the speed (delay) of operation, Hence it is necessary to focus the critical path delay of an multiplier. Hence, the hybrid Compressor based multiplier is proposed to optimize the delay of multiplier as compared to the existing methods.

### Hybrid technique

**Figure 3:**
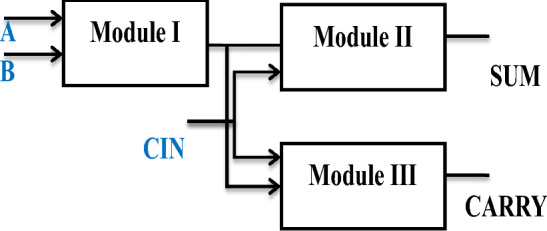
Block diagram for hybrid adder^8^.

Using more than one logic structure (styles) to design the Module of a system is known as hybrid technique. There are two types of logic styles are followed in the hybrid technique. They are (1) Homogeneous styles, it using same type of circuit style in all the stages is called Homogeneous structure. (2) Heterogeneous styles, it using the different type of circuit style in different stages is called Heterogeneous design. Consider an example of full adder. The full adder contains three modules such as two half adder unit and one OR gate module. By using two different logic styles in half adders and another one logic style is used in OR gate module. This type of structure is called as hybrid full adder. This hybrid technique will be optimizing the speed, size and power utilization of the circuit. The basic architecture of hybrid technique is shown in Fig. 3^16^.

### Hybrid compressor

In order to optimize the delay and size of multiplication architecture, it incorporates the above-mentioned Compressors in different modules (stages) of the multiplier architecture.

**Figure 4:**
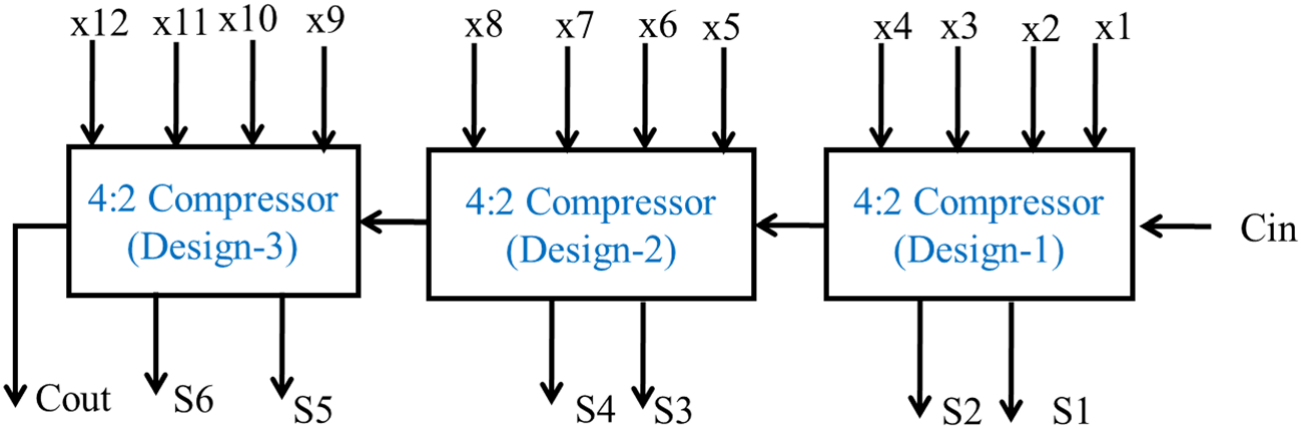
Architecture of 12:6 hybrid compressor.

**Figure 5:**
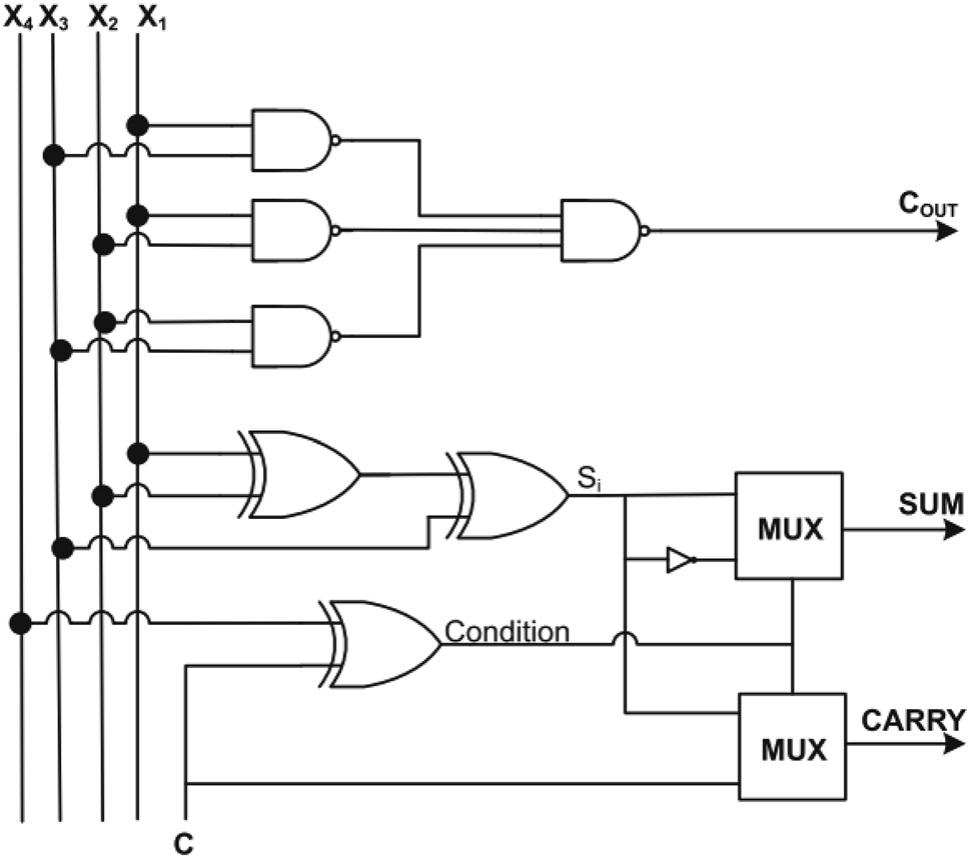
Architecture of 4:2 compressor (Design-1)^13^.

**Figure 6:**
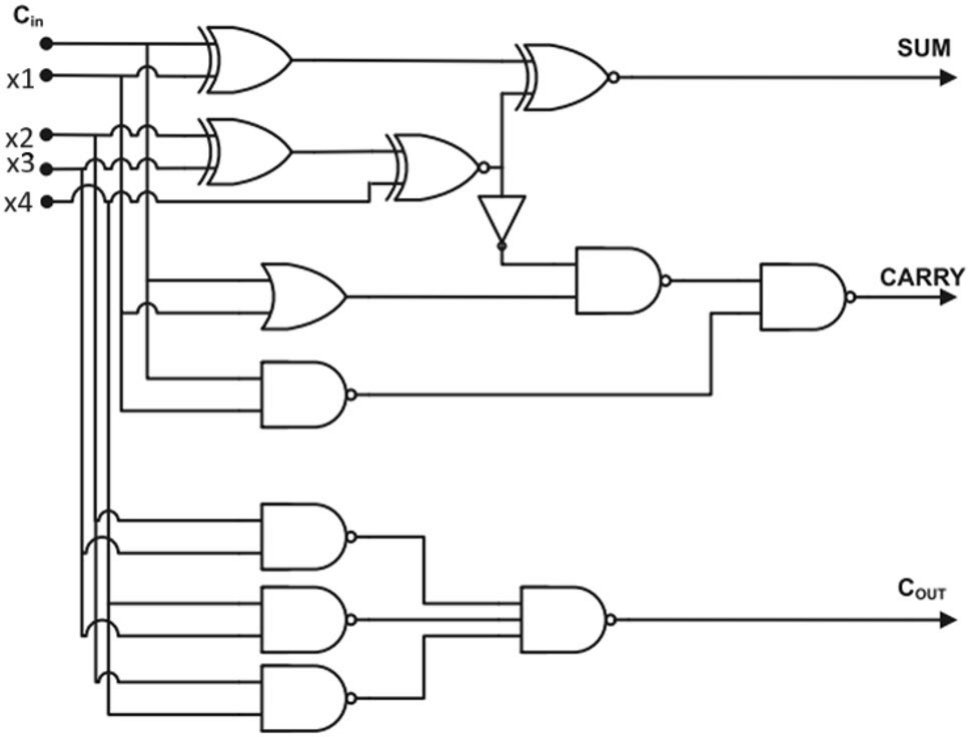
Circuit Diagram of 4:2 compressor (Design-2)^13^.

**Figure 7:**
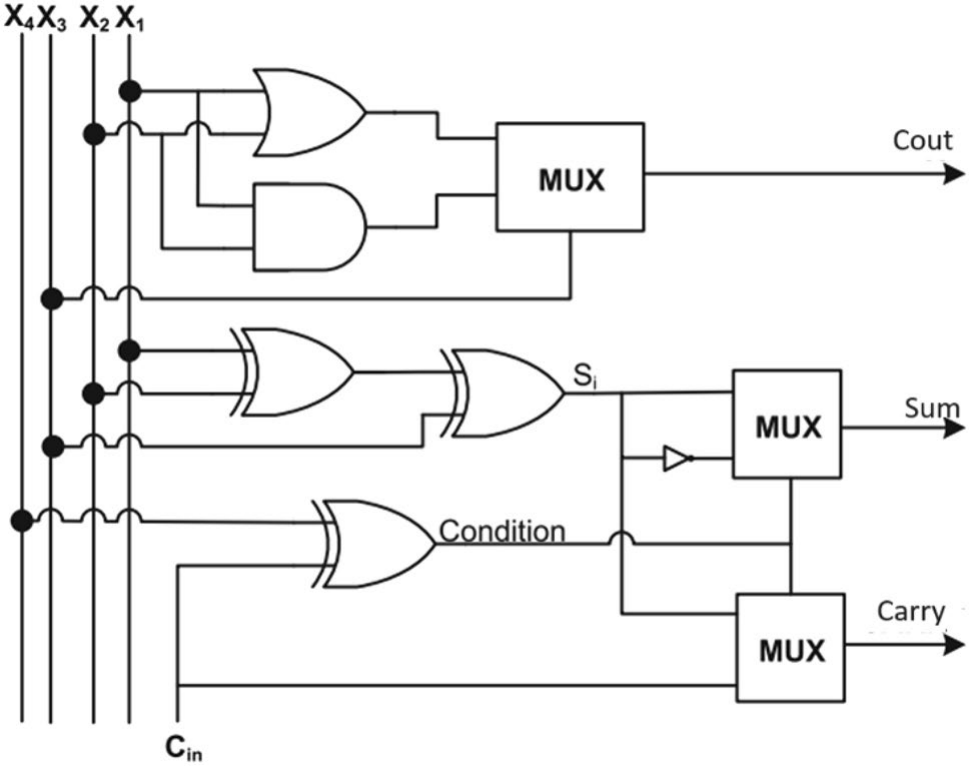
Architecture of 4:2 compressor (Design-3)^13^.

The architecture of 12:6 hybrid compressor is shown in Fig. 4. This compressor consists of 3 stages each with 4:2 compressor. The 4:2 compressor (design-1), 4:2 compressor (design-2) and 4:2 compressor (design-3) are used in stage1, stage2 and stage 3, respectively. Also shown in Figs. 5, 6 and 7 respectively.

The Fig. 5 and 6, 4:2 compressor was achieved considerable reduction in Area delay product and power delay product as compared to existing design^13^. It also is improved the delay of minimum 7% and maximum 12.5%^13^. Similarly, this compressor will be suitable for high performance multiplier and their relevant applications.

The refined logical relations for Design-1,2 and 3^13^ are

10 $${\text{Condition}} = {\text{X}}_{4}^{\prime } {\text{Cin}} + {\text{Cin}}^{\prime } {\text{X}}_{{4}} .$$

11 $${\text{S}}_{{\text{i}}} = {\text{X}}_{3}^{\prime } \left( {{\text{X}}_{{1}} \oplus {\text{X}}_{{2}} } \right) + {\text{X}}_{{3}} \left( {{\text{X}}_{{1}} \odot {\text{ X}}_{{2}} } \right).$$

12 $${\text{Sum}} = {\text{S}}_{{\text{i}}}^{\prime } \left( {{\text{X4}} \oplus {\text{Cin}}} \right) + {\text{Si}}\left( {{\text{X4}} \odot {\text{Cin}}} \right)$$

13 $${\text{Carry}} = {\text{Cin}}\left( {{\text{X4}} \oplus {\text{Cin}}} \right) + {\text{Si}}\left( {\text{X4 Cin}} \right)$$

14 $${\text{Cout}} = \left( {{\text{X1}} + {\text{X2}}} \right){\text{X3}} + {\text{X3}}\left( {{\text{X1}}\cdot{\text{X2}}} \right)$$

**Figure 8:**
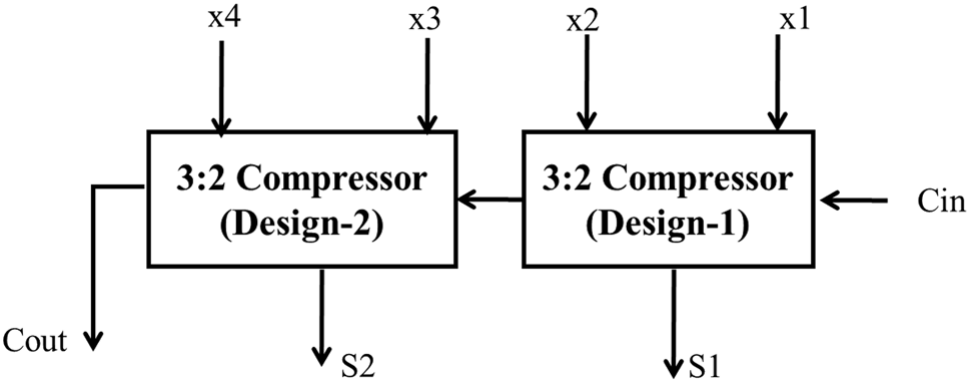
Architecture of 5:3 hybrid compressor.

**Figure 9:**
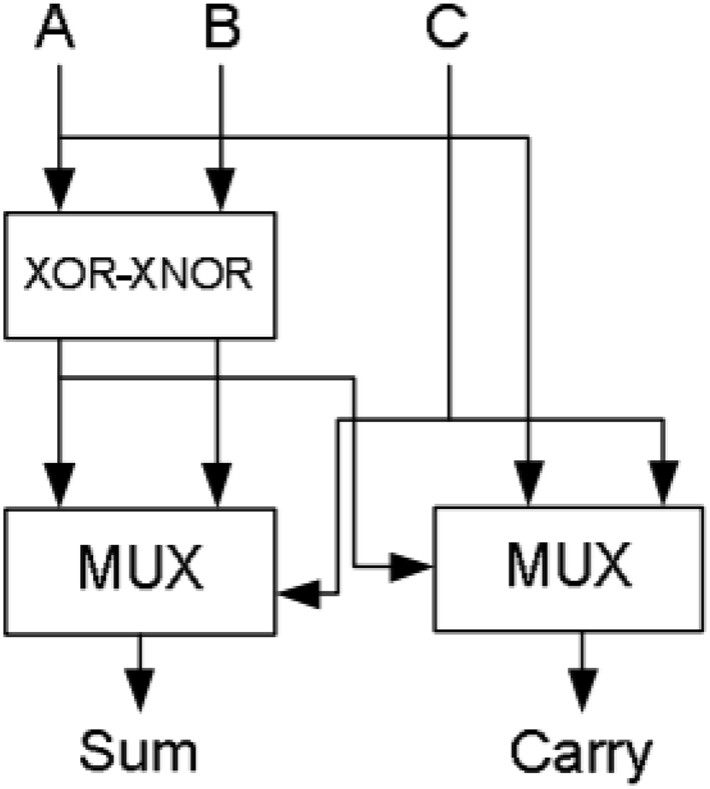
Architecture of 3:2 compressor (Design-1)^18^.

**Figure 10:**
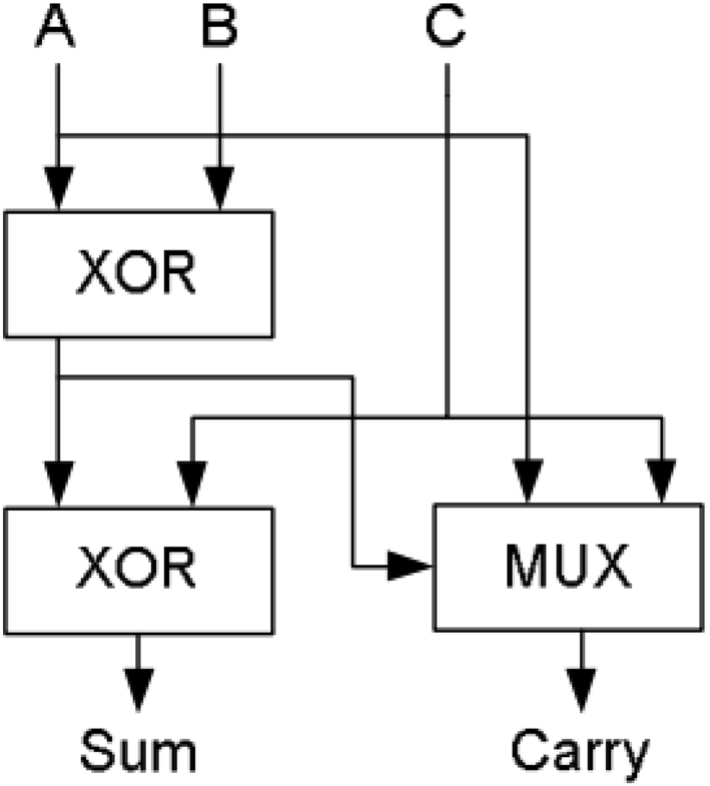
Structure of 3:2 compressor (Design-2)^18^.

The Architecture 5:3 hybrid compressor is shown in Fig. 8. This compressor consists of 2 stages each with 3:2 compressor. The 3:2 compressor (design-1) and 3:2 compressor (design-2) are used in stage1 and stage2 respectively. Also displayed in Figs. 9 and 10 respectively.

#### Proposed multiplication using hybrid compressor

**Figure 11:**
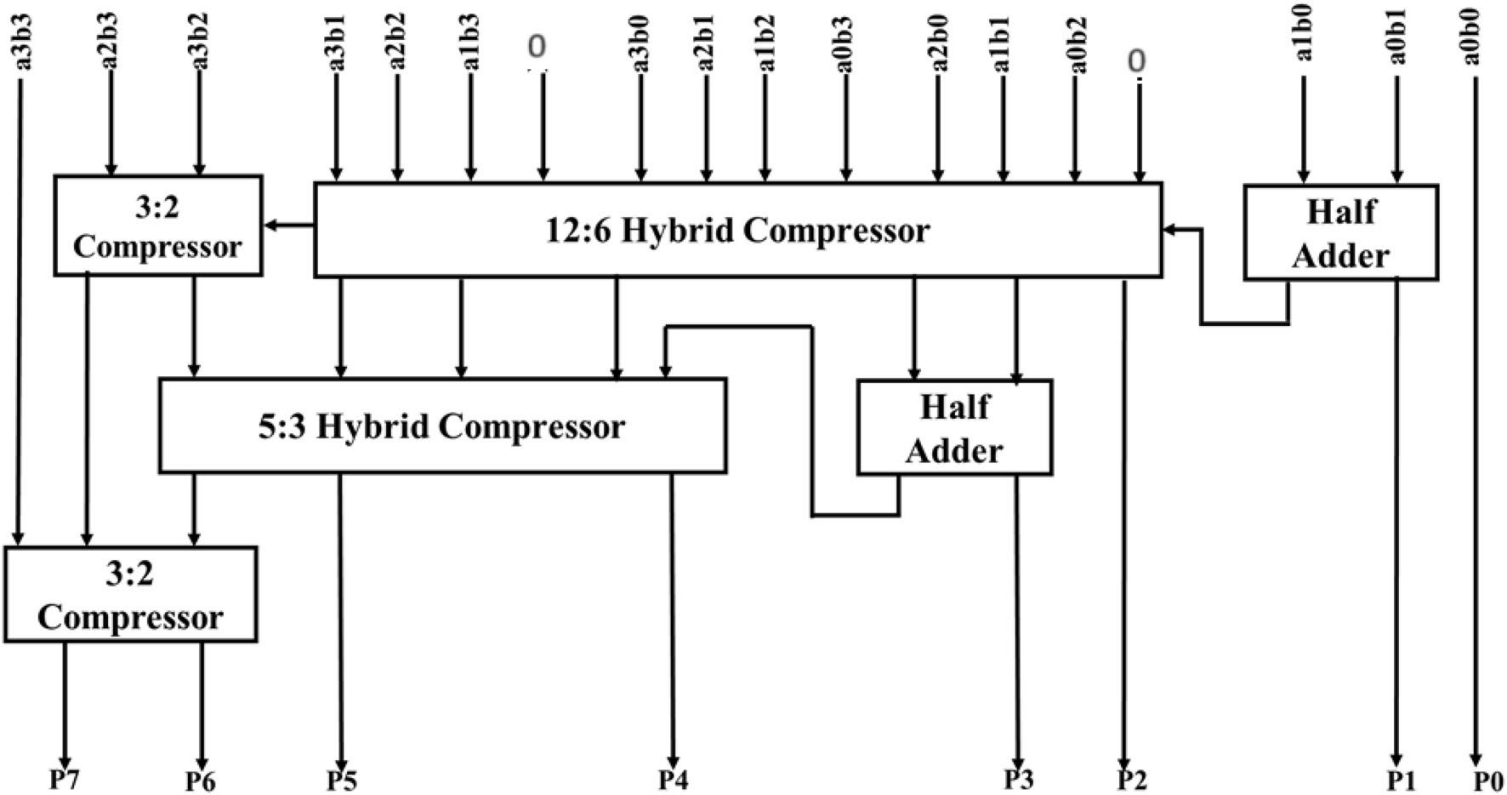
Structure of proposed 4*4 Multiplier using hybrid compressor.

The block diagram of the proposed hybrid compressor-based multiplier is displayed in Fig. 11. This structure is a 4 ∗ 4 multiplier. The partial products are computed with help of series of AND gates.

**Figure 12:**
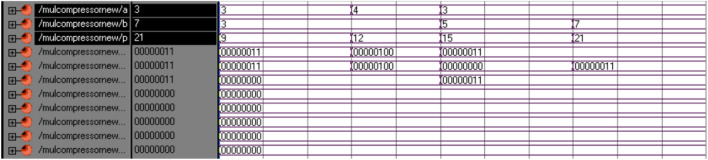
Simulation result of proposed hybrid compressor based multiplier.

Partial product computation is a 1st process of multiplier operation. Partial products are generated by multiplying each bit of multiplicand by multiplier. Consider an example of 4*4 Multiplication, Multiplier ‘A’ having 4 bits (A0 to A3) and Multiplicand ‘B’ having 4 bits (B0 to B1). In Computation of Partial product, the 1^st^ step is multiplying LSB of B(B0) with every bit of multiplier A (A0 to A3) and outputs are stored in C (C0 to C3) each with 4 bit. It generally represented as C0 = B0.A0(logical AND operation), C1 = B0.A1 and so on. Similarly, it multiplies B1, B2 and B3 bit with every bit of A in subsequence steps. The structure of partial product computation is displayed in Fig. 12.

The proposed hybrid compressor-based multiplier consists of three stages. In every stage, various-sized hybrid compressor and half adder are used. Namely, 12:6 compressor (combination of various design styles of 4:2 compressor) is used in the first stage (3 numbers of 4:2 compressor), 5:3 compressor (combination of various design styles of 3: 2 compressor) is used in the 2nd stage (two numbers of 3:2 compressor) and a one 3:2 compressor is used in the third stage (one number of 3:2 compressor).

## Performance analysis

**Figure 13:**
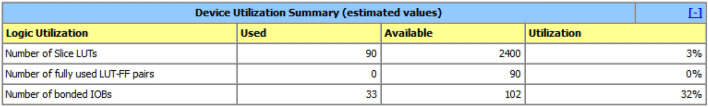
Device utilization for proposed multiplier using hybrid compressor.

The different techniques of multiplier, adder and compressor are deliberated in the “Review of adders”, “Proposed multiplier” & “Performance analysis” sections and proposed multiplier is elucidated in “Conclusion” section. Simulation of all multiplier architecture done in XILINX ISE (Integrated Software Environment). The Figs. 12 and 13 are display the input/output wave form and percentage of device utilizations of hybrid compressor-based multiplier respectively. The same is implemented in spartan6 Field programmable Gate Array (FPGA) device. All the multiplication technique are verified their input and output individually.

The synthesized results indicated that the delay, Number of Look Up Tables (LUTs) (Size), power consumption of several multiplier technique and it shown in the Table 1. The percentage of speed improvements in terms of delay for hybrid compressor-based multiplier is shown in Fig. 15 as compared to existing multiplier techniques.

**Table 1:** Simulation result of different multiplier with Spartan6 FPGA implementation.

S. No.	Name of the technique	Delay (ns)	No. of LUTs*	Power (uW)	ADP*	PDP*
1	Proposed Multiplier using hybrid compressor	13.75	83	58.25	1141.25	800.93
2	Hybrid Vedic multiplication (VM)^12^	14.81	86	55.63	1273.66	823.80
3	Hybrid multiplier based on carry select adder (CSELA)^8^	16.73	90	53.43	1505.7	893.88
4	Vedic multiplier using Han Carlson Adder (HCA)^12^	17.21	126	55.42	2168.46	953.77
5	Vedic multiplier using Ripple Carry Adder (RCA)^12^	17.33	108	53.51	1871.64	927.32
6	Revised Booth Multiplier (RBM)^8^	17.55	91	53.73	1597.05	942.96
7	Vedic Multiplier (VM) using Carry Look ahead Adder (CLA)^12^	19.23	113	54.93	2172.99	1056.3
8	Wallace tree multiplier (WTM)^8^	21.02	116	59.24	2438.32	1245.2
9	Array Multiplier (AM)^8^	21.43	84	52.52	1800.12	1125.5

*ADP, area delay product; PDP, power delay product; LUTs, look up tables.

The comparison shows the speed in terms of delay of the hybrid Compressor based multiplier is improved 35.83%, 34.58%, 21.65%, 28.49%, 20.65%, 20.10%, 17.81%, 07.15% as compared to Array Multiplication, Wallace tree multiplication, Booth Multiplier, Vedic Multiplier using Carry Look ahead Adder (CLA), Vedic Multiplication using Ripple Carry Adder (RCA), Vedic Multiplication using Han Carlson Adder (HCA), Hybrid Multiplier using Carry Select Adder (CSELA) and Hybrid Vedic Multiplier respectively.

Also, the Area delay product (ADP) of the hybrid compressor-based multiplier is enriched by 36.60%, 53.19%, 47.48%, 39.02%, 28.54%, 47.37%, 24.20% and 10.39% as compared to Array Multiplication, Wallace tree multiplication, Vedic Multiplication using Carry Look ahead Adder (CLA), Vedic Multiplier using Ripple Carry Adder (RCA), Booth Multiplication, Vedic Multiplier using Han Carlson Adder (HCA), Hybrid Multiplier using Carry Select Adder (CSELA) and Hybrid Vedic Multiplier respectively. As well as Power Delay Product (PDP) of the hybrid compressor-based multiplier is enhanced by 28.83%, 35.67%, 24.14%, 13.62%, 15.06%, 16.02%,10.39% and 2.77% as compared to Array Multiplication, Wallace tree multiplication, Vedic Multiplication using Carry Look ahead Adder (CLA), Vedic Multiplier using Ripple Carry Adder (RCA), Booth Multiplication, Vedic Multiplier using Han Carlson Adder (HCA), Hybrid Multiplier using Carry Select Adder (CSELA) and Hybrid Vedic Multiplication respectively.

**Figure 14:**
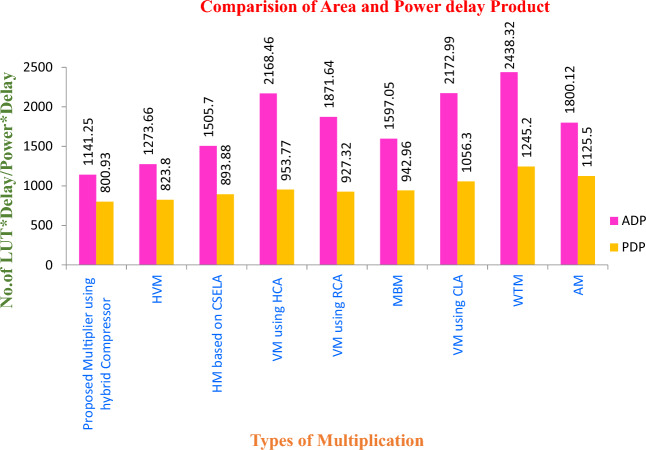
Analysis of ADP and PDP of various multiplier.

The Fig. 14 is displayed the Power Delay Product (PDP) and Area Delay Product (ADP) of different multiplication technique. The proposed compressor-based multiplier has a considerable improvement in PDP and ADP.

**Figure 15:**
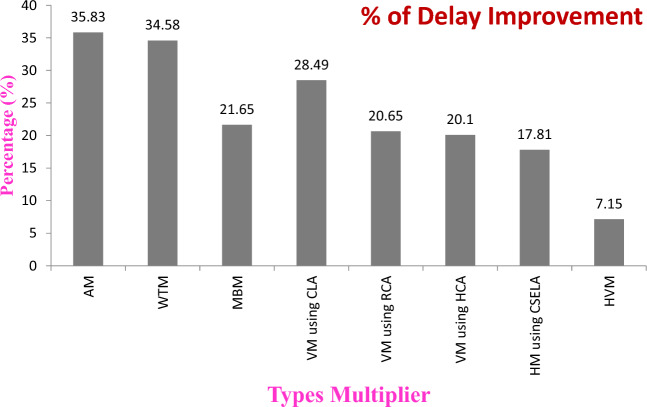
Percentage of delay improvements in Hybrid compressor-based multiplier as compared to other multiplier.

The comparison about delay improvement (%) of proposed multiplier using hybrid compressor is displayed in the chart as displayed in Fig. 15. Also delay of various multiplication techniques of research article is shown in Table 2. In that table it shows that the significant improvement delay is there in proposed multiplier using hybrid compressor.

**Table 2:** Delay analysis of 8*8 multiplier in existing article.

S. No.	Name of the multiplier in existing article	Delay (ns)	Area (No. of LUTs)	Power (uW)
1	High speed Vedic Multiplication^19^	23.64	192	51.78
2	Hybrid Vedic Multiplier^12^	14.80	86	55.70
3	Revised Vedic multiplier Design–2^15^	18.39	–	–
4	Revised Vedic Multiplier Design-1^15^	18.61	–	–
5	High Speed Hybrid Multiplier^8^	16.68	90	53.54
6	High Performance Multiplier^17^	20.70	92	50.17
7	Improved Binary Multiplication^18^	18.46	125	51.27
8	Proposed Multiplier using hybrid compressor	13.75	83	58.25

## Conclusion

In this investigation, Hybrid compressor-based multiplier architecture is experimented with various design styles of compressor. The results clearly indicated that the hybrid compressor-based multiplication has considerable improvement in speed of multiplication with reduced size as compared to existing multiplication technique. The proposed hybrid compressor-based multiplier architecture is successfully synthesized and simulated using Xilinx software and implemented on Field Programmable Gate Array (FPGA) boards. The synthesized result shows that the delay of proposed hybrid compressor-based multiplier is improved 35.83%, 34.58%, 21.65%, 28.49%, 20.65%, 20.10%, 17.81%, 07.15% as compared to Array Multiplication, Wallace tree multiplier, Booth Multiplier, Vedic Multiplier using Carry Look Ahead Adder (CLA), Vedic Multiplier Ripple Carry Adder (RCA), Vedic Multiplication using Han Carlson Adder (HCA), Hybrid Multiplier using Carry Select Adder (CSELA) and Hybrid Vedic Multiplier respectively. The comparative analysis of implementation results motivates authors to conclude that the proposed Hybrid compressors-based multiplier shall be a desirable choice for the implementation of high-performance signal^20^ and image processing^21^ and other related applications^22^.

## Data Availability

The datasets used and/or analyzed during the current study available from the corresponding author on reasonable request.
